# Differences in the Effects of Reading and Aerobic Exercise Interventions on Inhibitory Control of College Students With Mobile Phone Addiction

**DOI:** 10.3389/fpsyt.2022.797780

**Published:** 2022-03-01

**Authors:** Junyi Zhou, Lulu Wang

**Affiliations:** ^1^School of Physical Education and Sport Sciences, Fujian Normal University, Fuzhou, China; ^2^Provincial University Key Laboratory of Sport and Health Science, Fujian Normal University, Fuzhou, China; ^3^Key Laboratory of Kinesiological Evaluation General Administration of Sport of China, Fujian Normal University, Fuzhou, China

**Keywords:** inhibitory control, reading, aerobic exercise, college student, mobile phone addiction

## Abstract

Although many previous studies have shown that short-time moderate-intensity aerobic exercise can improve one's inhibitory control, some researchers suggested that its effect on inhibitory control is small. Meanwhile, some studies have shown that reading has a positive effect on inhibitory control. Since many studies examining the effect of exercise on inhibitory control used reading as a filler task, it is important to compare their effects. The present study used the antisaccade task as a tool to examine the differences in the effects of aerobic exercise and reading on inhibitory control of college students with mobile phone addiction. Thirty healthy college students with mobile phone addiction (range: 17–20 years, mean: 19.2 years) took part in the experiment. Participants were randomly assigned to an aerobic exercise group and a reading group. For the aerobic exercise group, participants were asked to perform moderate-intensity aerobic exercise for 15 min. For the reading group, participants were asked to sit quietly and read articles from newspapers for 15 min. Each participant's inhibitory control was examined pre- and post-intervention using the antisaccade task. In the antisaccade task, they have to direct their gaze toward the mirror image location of the target appearing parafoveally as quickly and as accurately as possible. The results showed significant main effects of Time (pre-test vs. post-test) on antisaccade latency and error rate. More importantly, a significant interaction of Time (pre-test vs. post-test) and Group (aerobic exercise vs. reading) was found on antisaccade latency. Specifically, the antisaccade latencies in the post-test were significantly shorter than those in the pre-test for the reading group, but the antisaccade latencies in the post-test and pre-test were comparable for the aerobic exercise group. The results of the present study imply that although both exercise and reading have effects on inhibitory control of college students with mobile phone addiction, the effect of reading may be somehow superior to exercise. Moreover, the current results also imply that researchers should be cautious when using reading as a filler task in future studies regarding the effect of aerobic exercise. The limitations of the present study were discussed.

## Introduction

Nowadays, the mobile phone is more than just a tool for communication. People use the Internet for consumption, entertainment, work, and study. It can be said that this is a mobile-phone-centered digital age. There are vast numbers of mobile phone users in the world (1, 2). Likewise, according to the latest Statistics of the 48th Statistical Reports on Internet Development in China, as of June 2021, the number of mobile phone Internet users in China reached 1.007 billion. 71.6% of people in China have access to the Internet via their mobile phones (3). Among these mobile phone users, college students make up a substantial portion. For college students, using mobile phones for communication, shopping, entertainment, and study is part of their daily life. However, although mobile phones bring much convenience and fun to college students, they also make college students suffer a large number of negative outcomes (4, 5). Among them, mobile phone addiction is one of the most common negative outcomes (6, 7). Mobile phone addiction would lead to poorer self-regulation, attentional control, and inhibitory control than normal individuals (8). Research has shown that about 21.3% of Chinese college students suffer from mobile phone addiction (9). Meanwhile, mobile phone addiction will cause significant impairments in academic performance and social relationships for college students (10–12). More seriously, college students with mobile phone addiction would suffer from severe mental disorders such as insomnia, anxiety, depression, and suicidal ideation (1, 2). Therefore, how to reduce the mobile phone addiction of college students has become the focus of researchers in recent years.

Inhibitory control is one of the three core components of executive functions (13). Inhibitory control enables individuals to focus on task-relevant information and suppress task-irrelevant information. Besides, inhibitory control helps individuals to control their emotions and behaviors (14). Of the three core components of executive function (i.e., inhibitory control, working memory, and cognitive flexibility), inhibitory control is most directly related to one's addiction or health behaviors. Study have shown that inhibitory control is a significant predictor of health behaviors (15). Neurobiological studies have shown that there is a intimate relationships between the circuits underlie inhibitory control and circuits disrupted by addiction behaviors. In fact, study do show that inhibitory control deficit is one of the critical factors contributing to mobile phone addiction (16). Previous studies have shown that individuals with higher levels of mobile phone addiction tend to have poorer inhibitory control, and increased inhibitory control is associated with a lower level of mobile phone addiction (16–18). Given the intimate relationship between inhibitory control and mobile phone addiction, we believe that it is important to explore effective ways to improve the inhibitory control function of college students with mobile phone addiction.

Moderate-intensity aerobic exercise refers to exercise with an intensity of 50–70% of one's maximum heart rate. Moderate-intensity aerobic exercise is deemed to be the most effective way to improve one's inhibitory control (19, 20) which were supported by many previous studies (21–24). Some studies found that 20 min of moderate intensity aerobic exercise can improve participants' performances on Stroop task (24, 25). More recently, researchers found that even 10 min moderate intensity aerobic exercise can improve participants' performance on antisaccade task (23). A newly published study conducted by Fan et al. (22) is most relevant to the present study. They found that 30 min acute aerobic exercise improved participants' performances on Go/no-go task. However, no significant improvement was found on participants' performances on Flanker task. However, there are some potential limitations in previous studies. **First**, most of the participants recruited in previous studies (except Fan et al.'s) were normal young adults rather than individuals with mobile phone addiction. Therefore, it remains unclear whether the results of previous studies could be generalized to individuals with mobile phone addiction. **Second**, previous studies used some cognitive tasks such as the Flanker task, Stroop task, and Go/no-go task to assess inhibitory control. However, some researchers argued that these tasks used to evaluate the inhibitory control require not only inhibitory control but also some other functions such as language, visual perception (Stroop task), and even perceptual-motor skill (Flanker task) (23, 26).Therefore, task involving both executive and non-executive components may not be able to detect subtle executive changes caused by short-time exercise. **Third**, in the study of Fan et al., which is the most relevant study of the present study, they assigned only 5 participants in each of the three groups of various intensity levels. This may result in somewhat less statistical power. Moreover, in Fan et al.'s study, the inhibitory control was assessed using the Flanker task and Go/no-go task. Yet, the pattern of the results obtained from the two tasks is incongruent. Therefore, the effect of moderate-intensity aerobic exercise on inhibitory control needs to be confirmed by further studies.

Furthermore, two studies using exercise interventions were brought to our attention. Wang et al. (27) used Wisconsin Card Sorting Test to investigate the effect of moderate-intensity aerobic exercise on executive function. Young adult participants were assigned to an aerobic exercise group or a reading control group. They failed to find the effect of the aerobic exercise effect on executive function. Heath et al. (28) used antisaccade task to investigate the effect of 10-min aerobic exercise with various intensities on executive function. Similarly, participants were assigned to exercise groups or reading control group. They found that antisaccade reaction times reduced significantly after aerobic exercise. Yet, the directional errors of antisaccade did not differ between exercise and control groups. These two studies both employed aerobic exercise as interventions and reading as filler tasks for participants in the non-exercise control group. Meanwhile, both studies obtained a null effect (either complete or partial). There are two possible reasons for these null effects. First, moderate-intensity aerobic exercise has no significant enhancement of executive function (or at least some aspects of it), as some previous studies have found (29–31). Second, reading as a filler task could improve executive function to some extent and act as a potential confound. For the first possibility, many previous studies have examined it extensively. Most of them revealed that aerobic exercise can improve executive function. Additionally, two meta-analysis studies showed that aerobic exercise has a small but positive effect on executive function (32, 33). Therefore, the first possibility is very unlikely. However, few previous studies have examined the second possibility. Previous studies do find that reading as a mean of cognitive activities is significantly associated with cognitive functions such as executive function (34–37). Although the above studies indicate a positive correlation between reading and executive function, the acute effect of reading remain unclear. Considering that reading was used as a filler task for participants in control groups in many studies regarding the effect of exercise on executive function. It would be necessary to examine the effect of reading on inhibitory control.

Therefore, the goal of the present study was to examine the potential differences in the effects of aerobic exercise and reading on inhibitory control by antisaccade task. The antisaccade task is a well-known experimental paradigm that is used to examine inhibitory control (23, 38, 39). In a typical antisaccade task, participants are instructed to fix on a central dot. Then, they have to direct their gaze toward the mirror image location of the target appearing parafoveally as quickly and as accurately as possible. Based on previous studies, we predict that both moderate aerobic exercise and reading can improve inhibitory control of college students with mobile phone addiction, and the effects of aerobic exercise and reading on inhibitory control of college students with mobile phone addiction are comparable. Specifically, we hypothesized that (1) participants would exhibit shorter antisaccade latency and lower saccade error after either aerobic exercise or reading intervention, and (2) there were no significant differences in these three measures between participants who received aerobic exercise and reading interventions.

## Method

### Ethics Statement

This study was approved by the Ethical Committee of the Fujian Normal University. All participants provided their written informed consent to participate in this study. This study was performed in full compliance with the Declaration of Helsinki.

### Participants

The G^*^Power tool (40) was used to calculate the sample size in the present study. Statistical Power analysis was conducted based on the reported effect size of aerobic exercise on antisaccade latency [mean Cohen's *d* = 1.43, from (28)]. The result indicated with an alpha level of 0.05, at least 24 participants are required to get a Power of 0.80 (*n* = 12 in each group). Therefore, we recruited 30 college students with mobile phone addiction from Fujian Normal University to participate in our experiment, which met the requirements of statistical power for replicating previous results. We used Mobile Phone Addiction Tendency Scale for College Student (MPATS) to screen participants. The MPATS consists of 16 items, each rated on a 5-point Likert scale, total of 80 points. A participant with a total score of 48 or above was classified as mobile phone addict (41). All these 30 participants scored over 48. Their ages and gender ranged from 17 to 20 years, with an average of 19.2 ± 0.88 years. The aerobic exercise group consisted of 15 participants (12 females and 3 males). The reading group consisted of 15 participants (11 females and 4 males). Each participant was paid ¥50 for their participation.

### Apparatus

The antisaccade task was programmed in Experimental Builder (SR Research Ltd.). The materials were presented on a 17-inch DELL PC laptop (DELL VOSTRO 15; 149 resolution: 1,920 ×1,080 pixels; refresh rate: 150 Hz). Stimulus were displayed in black (RGB: 0, 0, 0) on a gray background (RGB: 153, 153, 153). Participants were seated at a viewing distance of ~58 cm from the computer monitor. A chin rest was used to stabilize the participants' heads. Participants viewed stimulus binocularly while only their right eyes were monitored. Their eye movements were recorded using an Eyelink Portable Duo eye-tracking system with a sampling rate of 500 Hz.

### Procedure

**Figure 1:**
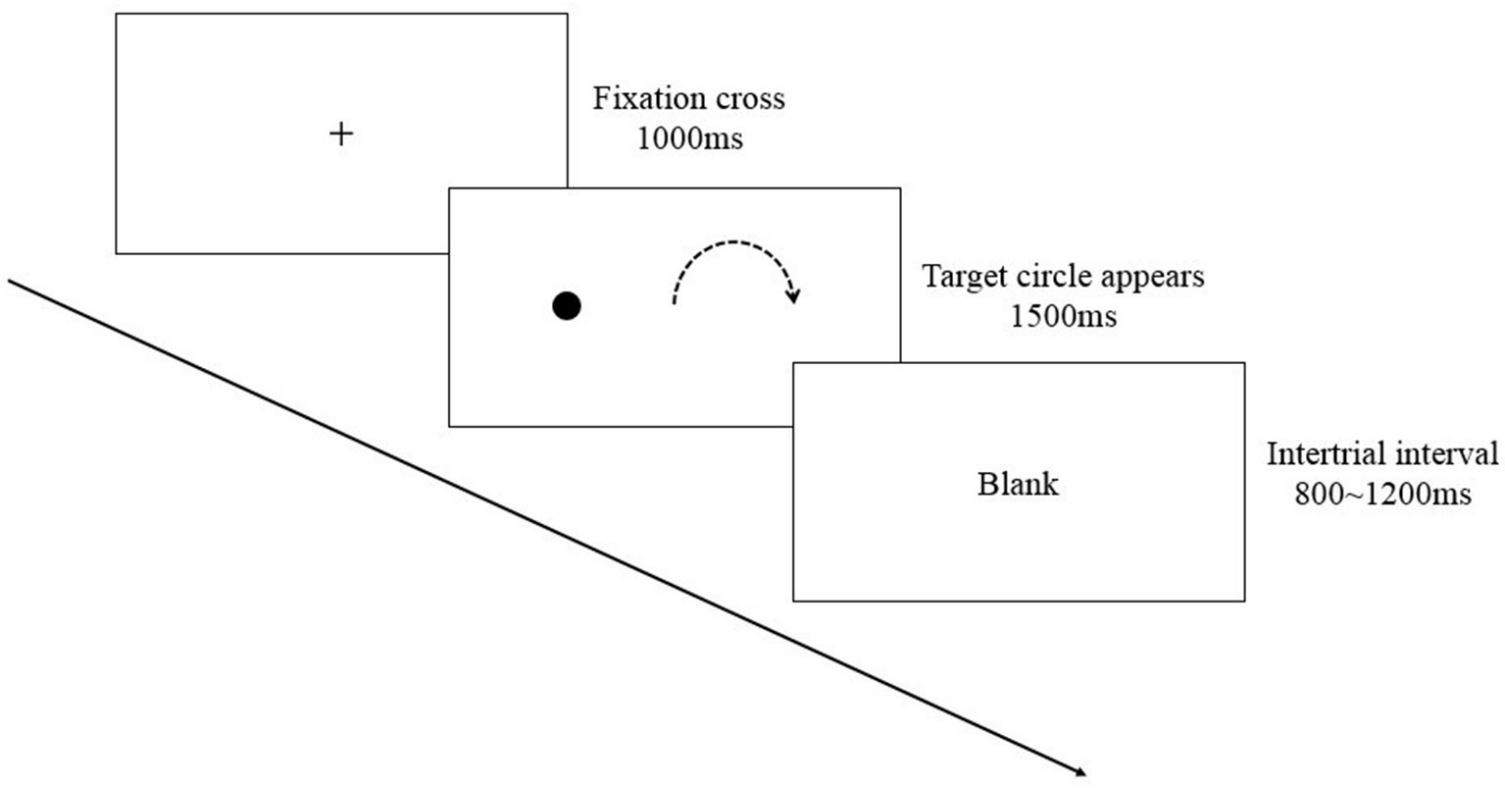
The illustration of the antisaccade task.

The experimental design of the present study is a two-factor mixed design with Group (aerobic exercise vs. reading) as a between-subject factor and Time (pre-test vs. post-test) as a within-subject factor. Thirty Participants were randomly assigned into two groups. For the aerobic exercise group, participants were asked to perform the moderate-intensity aerobic exercise using a bicycle ergometer (Ergoline, Germany) for 15 min. Participants' heart rates were monitored using a heart rate sensor (Polar, Finland) to ensure they were exercising at a moderate intensity. For the reading group, participants were asked to sit quietly and read articles from newspapers (which do not contain any pictures) for 15 min. The initial power of the bicycle was set to 50 W. Participants were asked to limited the revolution speed between 55–65 r/m. The resistance would be then adjusted to make sure each participant reach 60–70% of their maximum heart rate. Each participant's executive function was examined pre- and post-intervention using the antisaccade task. The antisaccade task comprised 75 trials. Five of them were practice trials. Each trial began with a fixation cross (1° ×1°) at the center of the screen displayed for 1,000 ms. Then, the target circle (1.2° ×1.2°) was displayed with an eccentricity of ±10° of visual angle in the horizontal plane for 1,500 ms (35 trials for each side), followed by an intertrial interval randomly varied between 800 and 1,200 ms. Participants were instructed to fixate at the cross to ensure that they were looking at the center of the screen when the target appeared peripherally. They were also instructed to direct their gaze toward the mirror image location of the target appearing parafoveally as quickly and as accurately as possible (see Figure 1). Participants were tested individually in a quiet room. After reading the experimental instructions and a brief description of the apparatus, the chair was adjusted to make them feel comfortable, and the eye tracker was calibrated using a nine-point calibration and validation procedure. The maximal error of validation was below 0.5° in the visual angle. At the beginning of each trial, a black circle (0.5° ×0.5°) was presented on the center of the computer screen as drift correction. Once the participant successfully fixated on the black circle, the following stimuli were displayed. The antisaccade task lasted about 12 min.

### Statistical Analysis

Data Viewer (SR Research Ltd.) was used to analyze the raw eye movement data. To ensure the including eye movement data are qualified, the following criteria for inclusion were adopted in analyses (42). (1) Saccades with a latency between 80 and 800 ms. (2) Saccade duration must be larger than 25 ms, and (3) saccade amplitude must be >3°. This resulted in a loss of ~10% of the trials. Based on these criteria, the following saccade measures were derived: (1) Antisaccade latency, which was defined as the time elapsed from the onset of the target to the onset of the first saccade toward the mirror image location of the target after target onset. (2) Error rate, the probability that participant wrongly executes a prosaccade instead of an antisaccade.

## Results

**Figure 2:**
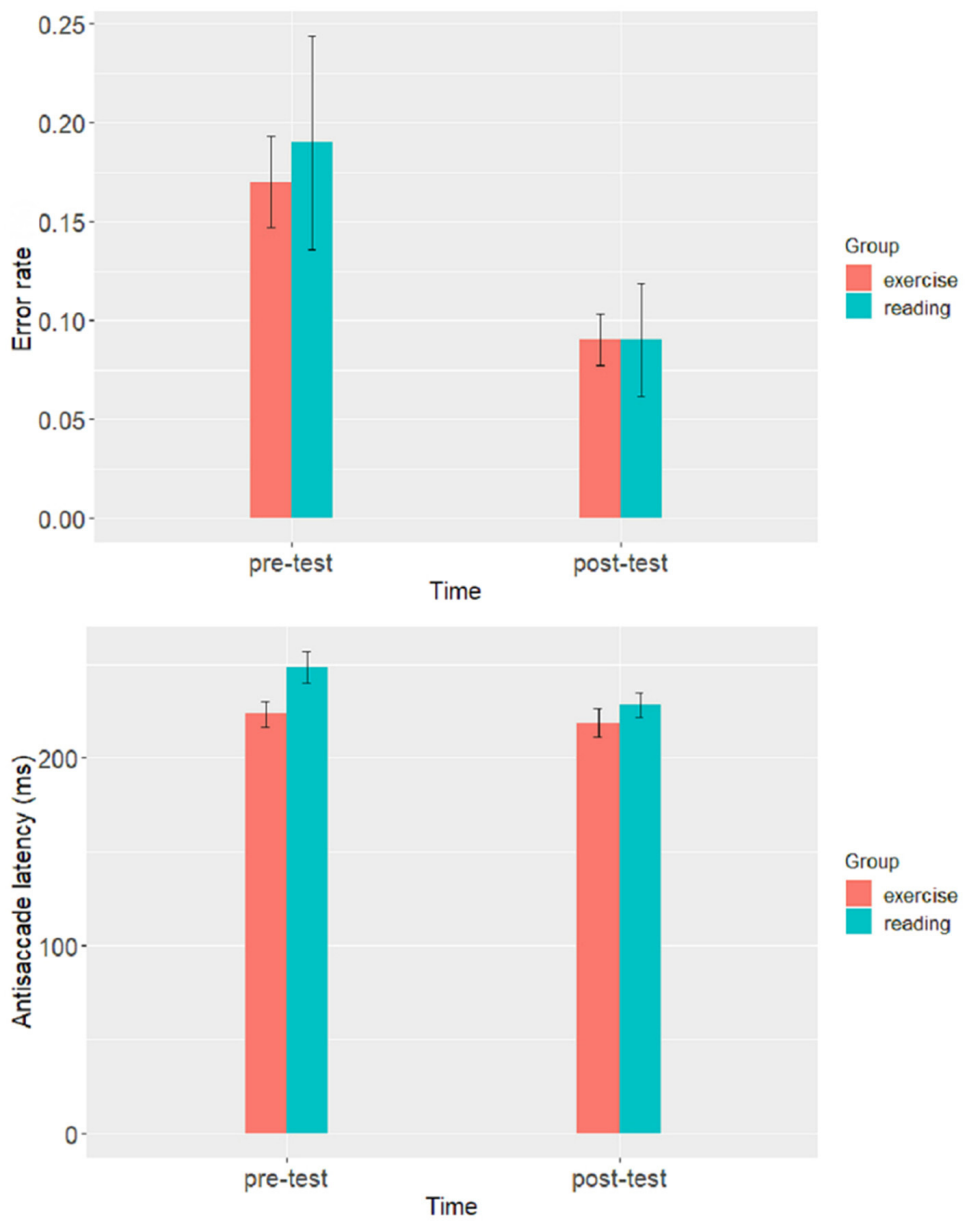
Differences in saccadic eye movement measures in pre- and post-test antisaccade task for two groups of students.

Eye movement measures of two groups in pre- and post-test antisaccade task are reported in Table 1. A 2 (Group: aerobic exercise vs. reading) by 2 (Time: pre-test vs. post-test) repeated measure ANOVA was performed for each dependent variable separately (see Figure 2 for detail).

**Table 1:** Mean (SD) of eye movement measures in pre- and post-test antisaccade task for two groups.

**Eye movement index**	**Reading group**		**Exercise group**	
	**Pre-test**	**Post-test**	**Pre-test**	**Post-test**
Error rate	0.19 (0.21)	0.09 (0.11)	0.17 (0.09)	0.09 (0.05)
Antisaccade latency	248.58 (33.09)	227.98 (25.07)	223.58 (26.38)	218.77 (28.19)

### Error Rate

The ANOVA revealed a significant main effect of Time, *F*_1, 28_ = 21.21, *p* < 0.001, $\eta_{p}^{2}$ = 0.43, but not Group, *F*_1, 28_ = 0.12, *p* = 0.733, $\eta_{p}^{2}$ = 0.004. The interaction of Time and Group was not significant, *F*_1, 28_ = 0.33, *p* < 0.573, $\eta_{p}^{2}$ = 0.01. The main effect of Time showed that the participants produced fewer error in post-test than in pre-test.

### Antisaccade Latency

The ANOVA revealed significant main effect of Time, *F*_1, 28_ = 16.13, *p* < 0.001, $\eta_{p}^{2}$ = 0.37, but not Group, *F*_1, 28_ = 3.01, *p* = 0.094, $\eta_{p}^{2}$ = 0.09. The interaction of Time and Group was significant, *F*_1, 28_ = 6.23, *p* < 0.019, $\eta_{p}^{2}$ = 0.18. The main effect of Time showed that the post-test antisaccade latencies were shorter than their pre-test counterparts. Further analysis revealed that the antisaccade latencies in post-test were significantly shorter than those in pre-test for participants in reading group, *t*_28_ = 14.38, *p* = 0.002. However, the antisaccade latencies in post-test did not differ from those in pre-test for participants in aerobic exercise group, *t*_28_ = 2.20, *p* = 0.160.

## Discussion

In the current study, we examine the differences in the effects of aerobic exercise and reading on inhibitory control of college students with mobile phone addiction by antisaccade task. As we hypothesized, the current results showed that participants would exhibit shorter antisaccade latency and lower saccade error after either aerobic exercise or reading intervention. However, the hypothesis that the effects of aerobic exercise and reading on inhibitory control were comparable was not fully supported by current results.

The results showed significant main effects of Time on antisaccade latency and error rate. These results suggest that both aerobic exercise and reading interventions can significantly improve the inhibitory control of college students with mobile phone addiction. This is consistent with previous studies finding that moderate-intensity aerobic exercise (21–24) or reading (34–37) can improve inhibitory control.

More importantly, the most critical and intriguing result of the present study is that a significant interaction of Time and Group was found on antisaccade latency. The antisaccade latencies in post-test were significantly shorter than those in pre-test for the reading group, yet, the antisaccade latencies in post-test did not differ from those in pre-test for the aerobic exercise group. This result suggests that reading has a positive effect on inhibitory control of college students with mobile phone addiction, and its effect was greater than that of aerobic exercise. This is consistent with previous studies concerning participating cognitive activities such as reading can improve one's inhibitory control (34–37). Yet, it is contrary to some other studies regarding the effect of aerobic exercise on inhibitory control (23, 28). These studies reported significant reductions in pre- to post-exercise antisaccade latency and non-significant changes in pre- to post-break (i.e., reading magazine) antisaccade latency. One thing we should note, however, is that the pattern of results varies across indicators. Specifically, we found a significant interaction on antisaccade latency, indicating that participants' antisaccade latency decreased after reading, whereas participants' antisaccade latency did not change significantly after aerobic exercise. However, no significant interactions were found on error rate. That is, a significant reduction in antisaccade latency after reading was not accompany with any significant change in error rate. Based on the explanation proposed by researchers in previous studies, this pattern indicates that participants did not decrease their post-test antisaccade latency at the cost of decreased accuracy (23, 28, 43). Additionally, we believe that this pattern of results may indicate that saccade latency is a more sensitive indicator which better reflects changes caused by intervention than error rate.

Previous evidence does indicate that reading can improve one's executive function (44, 45). There are several reasons why reading can improve inhibitory control. On the one hand, reading has some positive psychological effects. Pawlowski et al. (37) suggested that reading can improve performance on neuropsychological tasks. During the reading process, one must focus on the current visual input and suppress the interference from internal and external irrelevant information to absorb and comprehend reading materials. Therefore, inhibitory control plays an important role in the reading process, especially in inhibiting spontaneously generated information and behaviors within the individual. Thus, one's inhibitory control can be improved through reading. On the other hand, reading activates brain areas associated with inhibitory control. Previous neuroscience research has shown that some cerebral cortexes are involved in reading tasks, including: left medial extrastriate cortex, left middle temporal cortex, left frontal cortex, and left posterior temporal lobe (46). Meanwhile, researchers have revealed that inhibitory control associated with the execution of the antisaccade task involves the activations of prefrontal executive networks (47) and frontoparietal networks (23, 48). These studies above indicate that the reading process and inhibitory control both refer to some overlapping regions, supporting the view that reading can improve one's inhibitory control from the perspective of neuroscience. Yet, it is important to note that although the present study found an acute effect of reading, most of the previous studies only reported the chronic effect of reading. Considering that few previous studies have compared the chronic and acute effects of reading on executive functions or inhibited control functions, the potential differences between these effects are not clear. We should therefore be cautious when interpreting these results.

The null effect of aerobic exercise on antisaccade latency shows that exercise intervention does not significantly improve the inhibitory control of students with mobile phone addiction. This is consistent with previous studies which failed to find a significant effect of the exercise intervention on improving inhibitory control or cognitive functions (27, 28). However, it is inconsistent with previous studies showing that moderate-intensity aerobic exercise can improve inhibitory control (21–24). This discrepancy with previous literature may be due to some possible reasons. First, the duration of the exercise intervention in the present study may not be long enough. A meta-analysis study showed that the effect of short-time (<20 min) aerobic exercise on cognitive performance and executive control is generally small or negative while the aerobic exercise of more than 20 min has a positive effect (32). Second, the difference of participant populations in the present study and previous studies may contribute to the discrepancy. Although meta-analytic studies have shown the effect of short-time aerobic exercise on cognitive performance and executive control are generally small or negative, the previous study does find that 10-min moderate-intensity aerobic exercise can improve one's inhibitory control (23). Therefore, we believe that another possibility for the discrepancy is that the participant population in the current study differs from those in previous studies. Most of the participants in the previous studies were normal young adults or normal senior adults (22, 23, 26, 43), whereas the participants recruited for the present study were college students with mobile phone addiction, whose inhibitory control may differ from that of the normal population. Third, there are differences between the reading materials used in the current study and previous reading materials. In the previous studies, magazines and novels were used as reading materials (27, 28), but in the present study, newspapers are read. Serious newspapers covering daily global affairs were chosen as reading material to avoid causing emotional arousal which may influence one's inhibitory control (13). This difference might contribute to the discrepancy.

This result also suggests that there may be another possible explanation for the results of the two previous studies which did not find the effect of aerobic exercise on executive function (27, 28). That is, it may be because reading has an effect on improving executive function as aerobic exercise. Therefore, the current pattern of results supports the second possibility we raised at the beginning to explain the null effects of these two studies (i.e., reading as a filler task could improve executive function to some extent and act as a potential confound).

Another important issue is the causal relationship between mobile phone addiction and inhibitory control. Most of the previous studies focused solely on the relationship between mobile phone addiction and inhibitory control or examining the effect of some interventions on inhibitory control of populations with mobile phone addiction. However, the causal relationship between mobile phone addiction and inhibitory control has rarely been discussed in depth in these studies. One relevant literature may provide some inspirations for our understanding of the causal relationship between them. Gao et al. (16) examined the deficient inhibitory control problematic mobile phone use using Go/no-go task and electrophysiological technology. 20 problematic mobile phone users and 19 controls were included in this study. The results showed that problematic mobile phone users had a weaker NoGo P3 amplitude (which is related to inhibitory control) than controls on the mobile phone application background. It seemed that it was the presence of mobile phone-related stimuli or mobile phone use that caused the impairment of one's inhibitory control. Moreover, the authors suggested that the result might indicate that there is no general impairment of inhibitory control in problematic mobile phone users. The deficient inhibitory control appeared merely in mobile phone related background. Nevertheless, we should note that there was difference in inhibitory control between problematic mobile phone users and controls. Why the controls did not show lower P3 amplitude? Thus, does mobile phone addiction lead to the impairment in inhibitory control? Or are one with lower inhibitory control more likely to become addicted to mobile phones? We acknowledge that our study is not sensitive enough to answer this question; therefore, further psychobiological studies are needed to determine the causal relationship between mobile phone addiction and inhibitory control.

The findings of the present study shed some light on understanding the effects of short-time moderate-intensity aerobic exercise and reading on inhibitory control of college students with mobile phone addiction. The current results indicate that a single short-time reading can improve inhibitory control of college students with mobile phone addiction which extends previous research regarding that reading as a daily cognitive stimulation can improve executive function (34–37). Besides, considering that many previous studies examining the effects of various exercise interventions used reading as filler tasks for participants in control groups (23, 27, 28), some caution should be exerted when employing reading as a filler task in future studies. Additionally, the current study extends the findings of previous studies about the positive effect of reading on executive function of the normal population to a mobile phone addiction sample. Furthermore, the present study has some clinical and practical implications. The current results provide important supporting evidence on that reading is an effective way to improve the inhibitory control of people with mobile phone addiction. Although we do not know the causal relationship between mobile phone addiction and inhibitory control, the present study may imply that it is possible to help people with mobile phone addiction to reduce their levels of addiction by improving their inhibitory control. Hence, future research could test this view by examining the acute effects of reading on both the inhibitory control and level of addiction of people with mobile phone addiction. Moreover, many previous studies focused solely on the acute effects of aerobic exercise on inhibitory control while few studies focused on the acute effects of reading. The current study provides preliminary evidence for future research regarding the acute effects of reading on inhibiting control. Reading as a simple, convenient and economic activity, is less constrained by environment, such that it would be a promising way of intervention to improve one's inhibitory control and reduce one's level of mobile phone addiction.

Nevertheless, owing to the restriction of conditions, we should note that the present study has some limitations. For example, the majority of the current sample was female college students (23 out of 30) which makes it unclear whether the results could be fully generalizable to a wider range of populations with mobile phone addiction. In addition, the level of mobile phone addiction was assessed using a self-reported questionnaire in the present study, which may differ from the actual level of mobile phone addiction. Future studies could use some developed APPs to assess the participants' mobile phone usage more objectively and precisely. Additionally, there are only a reading group and an aerobic exercise group in the present study as we focus on examining the difference of effect on inhibitory control between reading and aerobic exercise. Future research should set a negative control group within which participants do nothing. By setting a baseline, the effects of reading and aerobic exercise on inhibitory control can be better assessed.

## Conclusion

In summary, the results of the present study suggest that reading can improve inhibitory control of college students with mobile phone addiction and its effect may be better than short-time moderate-intensity aerobic exercise. Overall, the present findings are enlightening for understanding the effects of reading and aerobic exercise on inhibitory control in the young mobile phone addicted population.

## Data Availability Statement

The original contributions presented in the study are included in the article/Supplementary Material, further inquiries can be directed to the corresponding author/s.

## Ethics Statement

The studies involving human participants were reviewed and approved by the Ethical Committee of the Fujian Normal University. Written informed consent to participate in this study was provided by participants or the participant' legal guardian/next of kin.

## Author Contributions

Both authors listed have made equal contribution to the work and approved it for publication.

## Funding

This research was supported by a grant from the Fujian Social Science Foundation (FJ2019B043), and by a grant from the Planning Project of Fujian Provincial Department of Education (JAS180075).

## Conflict of Interest

The authors declare that the research was conducted in the absence of any commercial or financial relationships that could be construed as a potential conflict of interest.

## Publisher's Note

All claims expressed in this article are solely those of the authors and do not necessarily represent those of their affiliated organizations, or those of the publisher, the editors and the reviewers. Any product that may be evaluated in this article, or claim that may be made by its manufacturer, is not guaranteed or endorsed by the publisher.
